# Bibliometric and visualized analysis of global research on microRNAs in gastric cancer: from 2013 to 2023

**DOI:** 10.3389/fonc.2024.1374743

**Published:** 2024-05-10

**Authors:** Xiaoqin Wang, Caihua Wang, Wenjin Han, Congmin Ma, Jiaru Sun, Tianmeng Wang, Zhaozhao Hui, Shuangyan Lei, Ronghua Wang

**Affiliations:** ^1^ Department of Pediatrics, the First Affiliated Hospital of Xi’an Jiaotong University, Xi’an, China; ^2^ School of Nursing, Xi’an Jiaotong University Health Science Center, Xi’an, China; ^3^ School of Nursing, Xi’an Vocational and Technical College, Xi’an, China; ^4^ School of Public Health, Xi’an Jiaotong University Health Science Center, Xi’an, China; ^5^ Department of Radiotherapy, Shaanxi Cancer Hospital, Xi’an, China

**Keywords:** miRNAs, gastric cancer, bibliometric analysis, Frontiers, CiteSpace

## Abstract

Gastric cancer (GC) imposes a heavy burden on global public health, and microRNAs (miRNAs) play a crucial role in the diagnosis and treatment of GC. Therefore, it is necessary to clarify the hotspots and frontiers in the field of miRNAs in GC to guide future research. A total of 2,051 publications related to miRNAs in GC from January 2013 to December 2023 were searched from the Web of Science Core Collection database. CiteSpace was used to identify research hotspots and delineate developmental trends. In the past decade, China, Nanjing Medical University, and Ba Yi were the most contributing research country, institute, and author in this field, respectively. The role of miRNAs as biomarkers in GC, the mechanism of miRNAs in the progression of GC, and the impact of the mutual effects between miRNAs and *Helicobacter pylori* on GC have been regarded as the research hotspots. The mechanisms of miRNAs on glucose metabolism and the application of the roles of circular RNAs as miRNA sponges in GC treatment will likely be frontiers. Overall, this study called for strengthened cooperation to identify targets and therapeutic regimes for local specificity and high-risk GC types, and to promote the translation of research results into clinical practice.

## 1 Introduction

GC is the fifth most common cancer and the fourth leading cause of cancer-related deaths around the world (1), posing a heavy burden on public health worldwide. It is reported that there were 1.1 million new cases of GC and 0.8 million new cancer-related deaths worldwide in 2020 (2), and its global burden will expected to increase to 1.77 million by 2040 (3). Moreover, a considerable number of GC patients are asymptomatic in the early stages and would not be diagnosed until the late stages, with a 5-year relative survival rate of 33% (4). Currently, the screening method with the highest detection rate for GC is endoscopy-based screening, but the invasiveness and high cost of gastroscopy can only make it cost-effective for a few countries in screening for the general population (3). In this context, it is urgent to explore noninvasive early screening methods and prognostic monitoring for GC.

MicroRNAs (miRNAs) are a group of non-coding RNAs encoded by the genome, with a length of approximately 22 nucleotides (5), which is stable *in vivo* and non-degradable during long-term storage *in vitro* (6–8). A growing number of studies reported that multiple miRNAs are abnormally expressed in GC cells (9–16), playing a crucial role in the diagnosis and treatment of GC. On the one hand, they can affect the proliferation, migration, invasion, and apoptosis of GC cells (9, 14, 15), serving as potential diagnostic and prognosis biomarkers for GC [eg., miR-21 (17), EV-miR-215-5p (18), miR-148 (19), miR-383-5p (20), and miR-421 (21)]. On the other hand, they can target the induction of DNA damage and cell death in sensitive and drug-resistant cells (9–13), regulating multi-drug resistance of GC cells [e.g., miR-200c (22), miR-214 (23), miR-135b-5p (24), miR-522 (25), and miR-95-3p (26)].

Considering the complexity of miRNAs related to GC and their mechanisms, reviewing and prospecting this research field is of great significance for providing new insights into the pathogenesis of GC and opening up new possibilities for early diagnosis and treatment strategies. There have been several reviews on the role of miRNAs in GC [for instance, expression, regulation, and function of exosome-derived miRNAs in cancer progression and therapy (27), the merging roles of miR-92/miR-145 in GC (28, 29), and clinical crosstalk between miRNAs and GC (30)], few studies have applied bibliometric analysis to the field of miRNAs in GC to describe its hotspots and research direction. Bibliometric analysis has become a mature statistical method that utilizes quantitative techniques to capture a large amount of bibliometric data through developed algorithms to summarize the knowledge structures and development trends in the research field (31). By forming networks of multi-level research components (such as countries, institutions, journals, and keywords), scholars can intuitively understand the literature characteristics, research hotspots, and development trends in this field.

This study aimed to summarize the current status, hotspots, development trends, and research frontiers in the field of miRNAs in GC over the past decade based on bibliometric and visual analysis, with a view to promoting research incubation and academic development.

## 2 Methods

### 2.1 Data source and retrieval strategies

The related publications on miRNAs in GC from January 1, 2013 to December 31, 2023 were retrieved from the Web of Science Core Collection (WoSCC) database. The retrieval strategies were as follows: TI= (“gastric” OR “stomach”) AND TI= (“cancer^*^” OR “tumor^*^” OR “tumour^*^” OR “neoplas^*^” OR “malignan^*^” OR “carcinoma^*^” OR “adenocarcinoma^*^”) AND TI=(“miR”) AND DOP= (2013-01-01/2023-12-31). Setting publication language to “English” and paper type to “article” or “review”, and excluding withdrawn publications, a total of 2,051 publications were retrieved and exported in the form of “Full Records and Cited References”. The above procedures were independently conducted by two researchers.

### 2.2 Data analysis

CiteSpace 6.2.R1 (64-bit) software, a visual analysis tool developed by Dr. Chaomei Chen (32), was used to map the current status, hotspots, and frontiers of research on miRNAs in GC. The parameter settings for CiteSpace software in this study were as follows: (a) timespan from January 2013 to December 2023, year per slice=1; (b) term source = title/abstract/author/keywords (DE)/keywords plus (ID); (c) node types = author, institution, country, keyword, reference, respectively; (d) selection criteria = the top 10% most cited or occurred items for each slice; (e) the other parameters were set to default values. Modularity Q value (Q) and Weighted Mean Silhouette value (S) are the two indicators showed in the upper left corner of the figures, generated by Citespace software to evaluate clustering effect. Q > 0.3 indicates that the division of clustering structure is significant. S > 0.5 is usually considered to be reasonable in the clustering results (33).

## 3 Results

### 3.1 Annual publications

**Figure 1:**
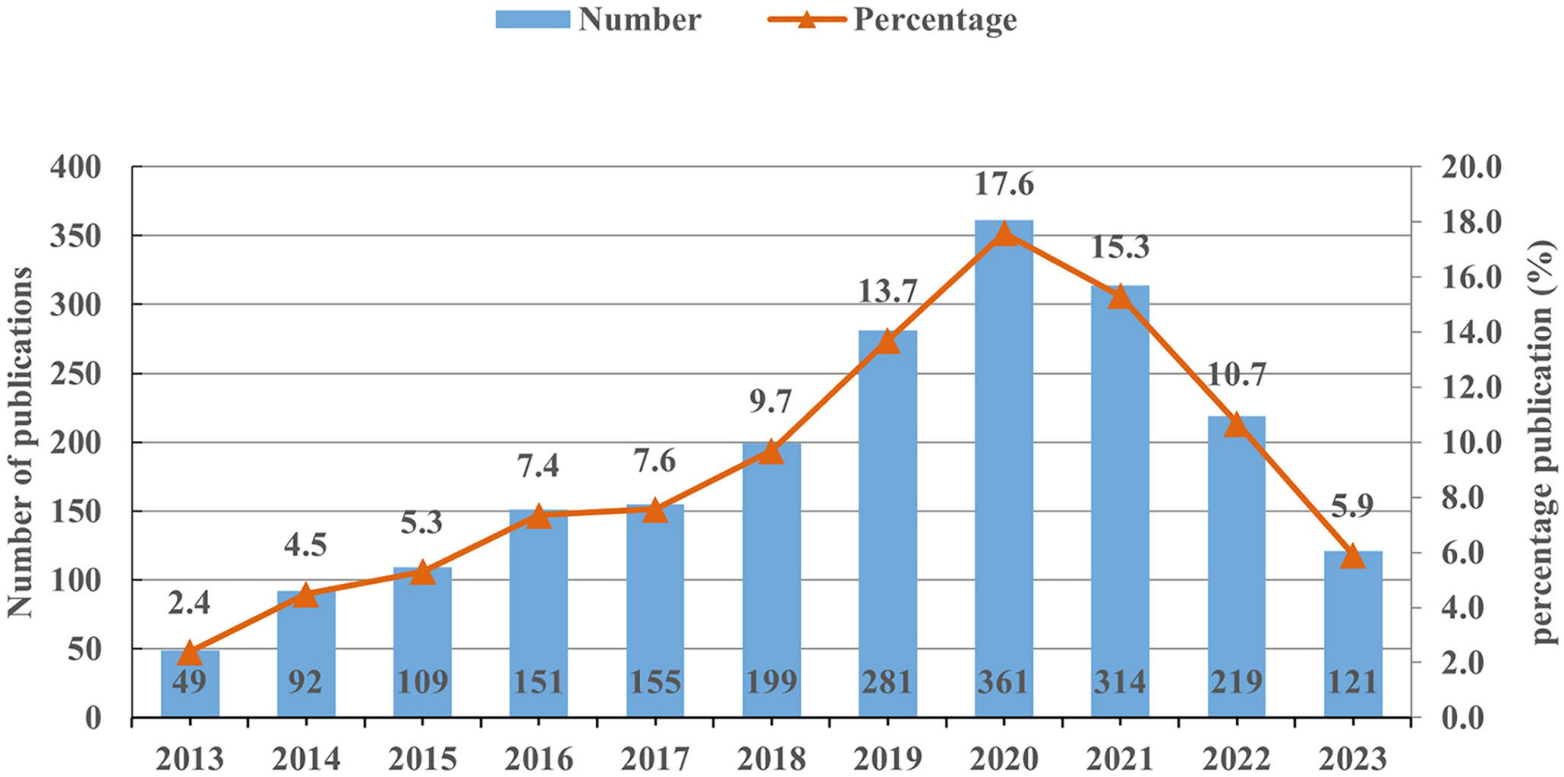
The annual number of publications on miRNAs in GC from 2013 to 2023.

A total of 2,051 publications on miRNAs in GC were included in this analysis. Overall, the number of publications in the past 11 years can be divided into three stages (**Figure 1**). In the first stage (2013–2017), the number of publications slowly increased with fluctuations. As more scholars focused on miRNAs in GC since 2017, the annual publication numbers showed a rapid increase from 155 to 361. While in the third stage (2020–2023), the number of publications began to decline, reaching 121 in 2023. Nevertheless, the average annual publication volume in the past three years remained at a high level of 218, indicating that researchers still have a high level of attention to this field.

### 3.2 Distribution of journals

It was found that papers included in this study were published in 380 journals. The list of top 10 productive journals showed that *OncoTargets and Therapy* ranked first (n=65, 3.17%) (**Supplementary Table S1**). *Cell Death & Disease* ranked seventh with the highest impact factor (IF=9.0, Q1). It is an online journal dedicated to promote diverse and integrated areas of experimental and internal medicine with cancer, immunity, neuroscience, and cancer metabolism, which seeks to encompass the breadth of translational implications of cell death. Among the top ten journals, 30% were from the England and 20% were from the US.

### 3.3 Collaboration analysis

#### 3.3.1 Country and institution collaboration analysis

**Figure 2:**
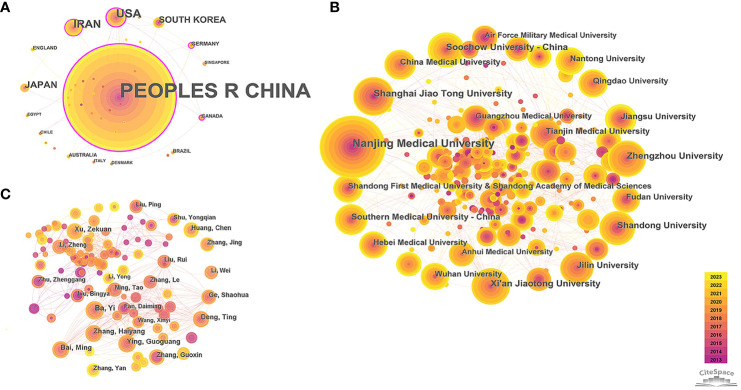
Collaboration network among research constituents. **(A)** Collaboration network of countries; **(B)** Collaboration network of institutions; **(C)** Collaboration network of authors. Note: The size of nodes indicates the number of publications for research constituents. The link between nodes shows the collaboration relationship, with thicker line indicating closer connection. The thickness of the purple ring around the node shows the strength of centrality (i.e., the thicker the purple ring, the greater its influence, and the closer its connection with other units), node with purple ring indicates its centrality > 0.1.

The country and institution cooperation networks can identify countries and institutions with a large number of publications and strong influence in the field of miRNAs in GC, and display their degree of cooperation. The 38 nodes and 45 links, 297 nodes and 640 links respectively constituted the country and institution collaboration network map (network density = 0.064 and 0.0146, respectively) (**Figures 2A, B**). China contributes the most publications (n=1924, 93.81%), far higher than the second ranked the United States (n=66, 3.22%) and the third ranked Iran (n=49, 2.39%). The top ten institutions in terms of output are all from universities in China, indicating that China is the leading country in this field (**Table 1**). Among the top 10 institutions with the highest output, Nanjing Medical University has the highest number of publications (n=185, 9.02%), followed by Shanghai Jiao Tong University (n=76, 3.71%) and Zhengzhou University (n=69, 3.36%).

**Table 1:** Top 10 countries, institutions and authors of publications.

No.	Countries	Count (%)	Centrality	Institution	Count (%)	Centrality	Authors	Count (%)	Centrality
1	Peoples R China	1924(93.81)	0.72	Nanjing Medical University	185(9.02)	0.09	Ba, Yi	22(1.07)	<0.01
2	United States	66(3.22)	0.69	Shanghai Jiao Tong University	76(3.71)	0.07	Bai, Ming	19(0.93)	0.01
3	Iran	49(2.39)	0.17	Zhengzhou University	69(3.36)	0.06	Ying, Guoguang	19(0.93)	<0.01
4	Japan	27(1.32)	<0.01	Xi'an Jiaotong University	67(3.27)	0.05	Zhang, Haiyang	19(0.93)	<0.01
5	South Korea	22(1.07)	<0.01	Soochow University - China	65(3.17)	0.03	Deng, Ting	18(0.88)	<0.01
6	Germany	5(0.24)	0.16	Shandong University	60(2.93)	0.02	Xu, Zekuan	18(0.88)	<0.01
7	Canada	4(0.20)	0.12	Jilin University	50(2.44)	0.01	Zhang, Guoxin	15(0.73)	<0.01
8	England	4(0.20)	<0.01	Southern Medical University - China	49(2.39)	0.02	Huang, Chen	15(0.73)	<0.01
9	Australia	4(0.20)	0.03	Jiangsu University	47(2.29)	<0.01	Ge, Shaohua	15(0.73)	<0.01
10	Brazil	4(0.20)	<0.01	China Medical University	47(2.29)	0.02	Zhang, Jing	14(0.68)	<0.01

It was found that 50% of the top 10 prolific countries have centrality exceeding 0.1, with China being the highest (centrality=0.72), reflecting its strongest cooperation with other countries. Germany (n=5, 0.24%) and Canada (n=4, 0.2%) have fewer publications in this field, but with relatively high levels of cooperation with other countries (centrality=0.16 and 0.12, respectively). However, the centrality of the top ten institutions is not ideal (centrality < 0.1), indicating academic exchanges between Chinese universities need to be further strengthened.

#### 3.3.2 Author collaboration analysis

The author collaboration network helps to explore influential authors and their cooperation in this field (**Figure 2C**), which is complex and contained 531 nodes and 1,315 links, with a network density of 0.0093. The top ten productive authors were all from China, which further strengthens the leading position of China in this field. Among them, Ba Yi, from the Peking Union Medical College Hospital and Tianjin Medical University Cancer Institute & Hospital, was the most contributing authors (n=22, 1.07%) (**Table 1**). Overall, the collaboration between authors was scattered (centrality ≤ 0.01), except for Bai Ming (centrality =0.01), and only a few scholars had established relatively close connections based on the working relationships of the same institutions.

### 3.4 Keyword co-occurrence analysis

**Figure 3:**
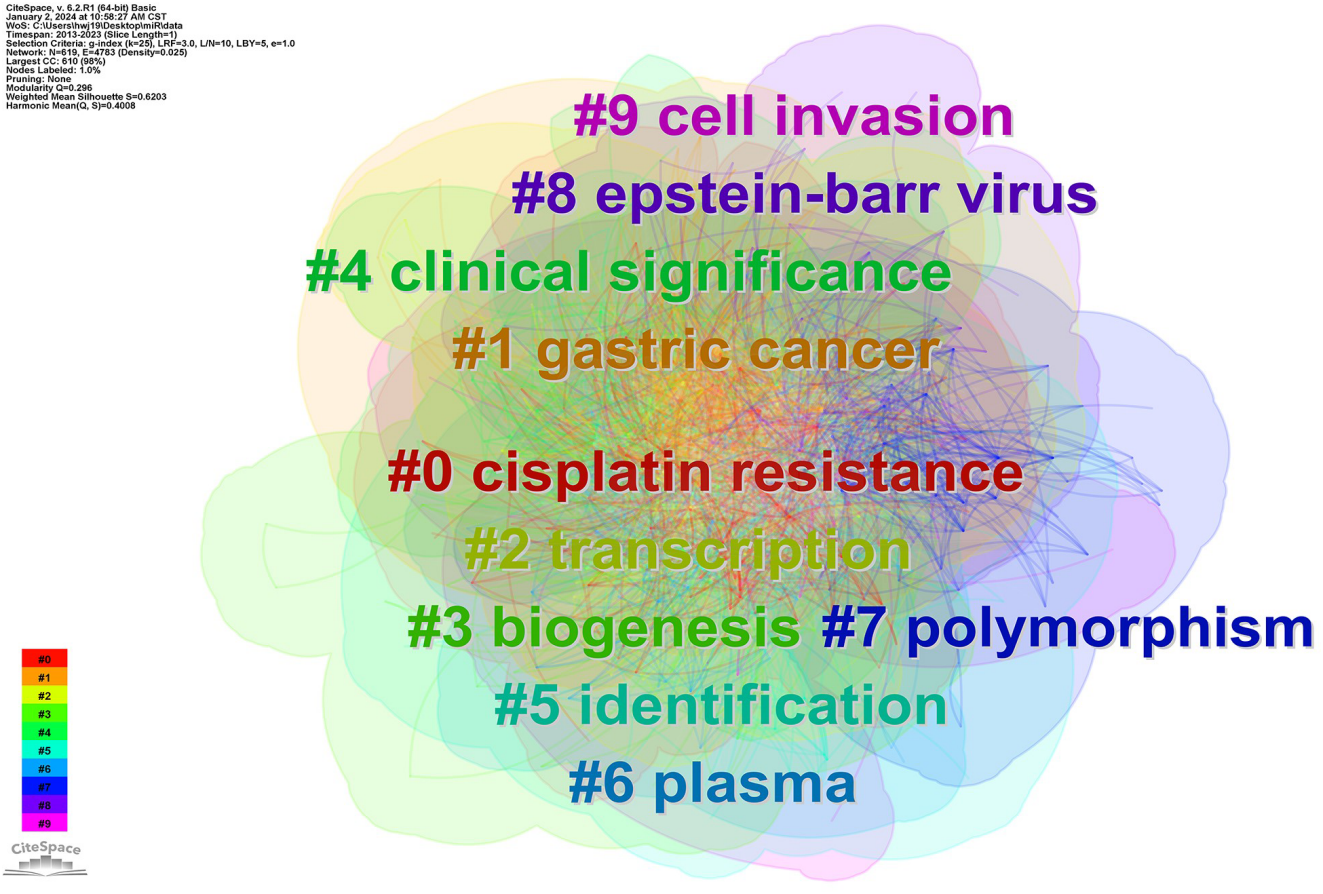
Cluster map of co-occurring of keywords on GC. Note: The links between nodes display publications with the same keywords (including synonyms), and their colors are consistent with the colors of keyword clusters.

Through the co-occurrence analysis of keywords, the research hotspots and the burst keywords in the field of miRNAs in GC have been reflected. After clustering co-occurrence keywords, 10 clusters were obtained (Q=0.296, S=0.6203) (**Figure 3**), including #0 cisplatin resistance, #1 gastric cancer, #2 transcription, #3 biogenesis, #4 clinical significance, #5 identification, #6 plasma, #7 polymorphism, #8 epstein-barr virus, and #9 cell invasion. In addition, the top 10 keywords in terms of quantity and centrality were listed (**Table 2**), which showed that the most frequent keyword was “gastric cancer” (n=1441), followed by “expression” (n=795), “invasion” (n=526), “proliferation” (n=477), and “metastasis” (n=464). And the top-ranked items by centrality were “identification” (centrality=0.11), “activation” (centrality=0.09), “overexpression” (centrality=0.07), “gene expression” (centrality=0.06), “breast cancer” (centrality=0.05).

**Table 2:** Top 10 keywords in terms of quantity and centrality.

No.	Keywords	Counts	Keywords	Centrality
1	gastric cancer	1441	identification	0.11
2	expression	795	activation	0.09
3	invasion	526	overexpression	0.07
4	proliferation	477	gene expression	0.06
5	metastasis	464	breast cancer	0.05
6	micrornas	401	biomarkers	0.05
7	growth	326	colorectal cancer	0.05
8	migration	313	lung cancer	0.05
9	progression	278	apoptosis	0.04
10	cells	261	poor prognosis	0.04

### 3.5 Burst keyword analysis

**Figure 4:**
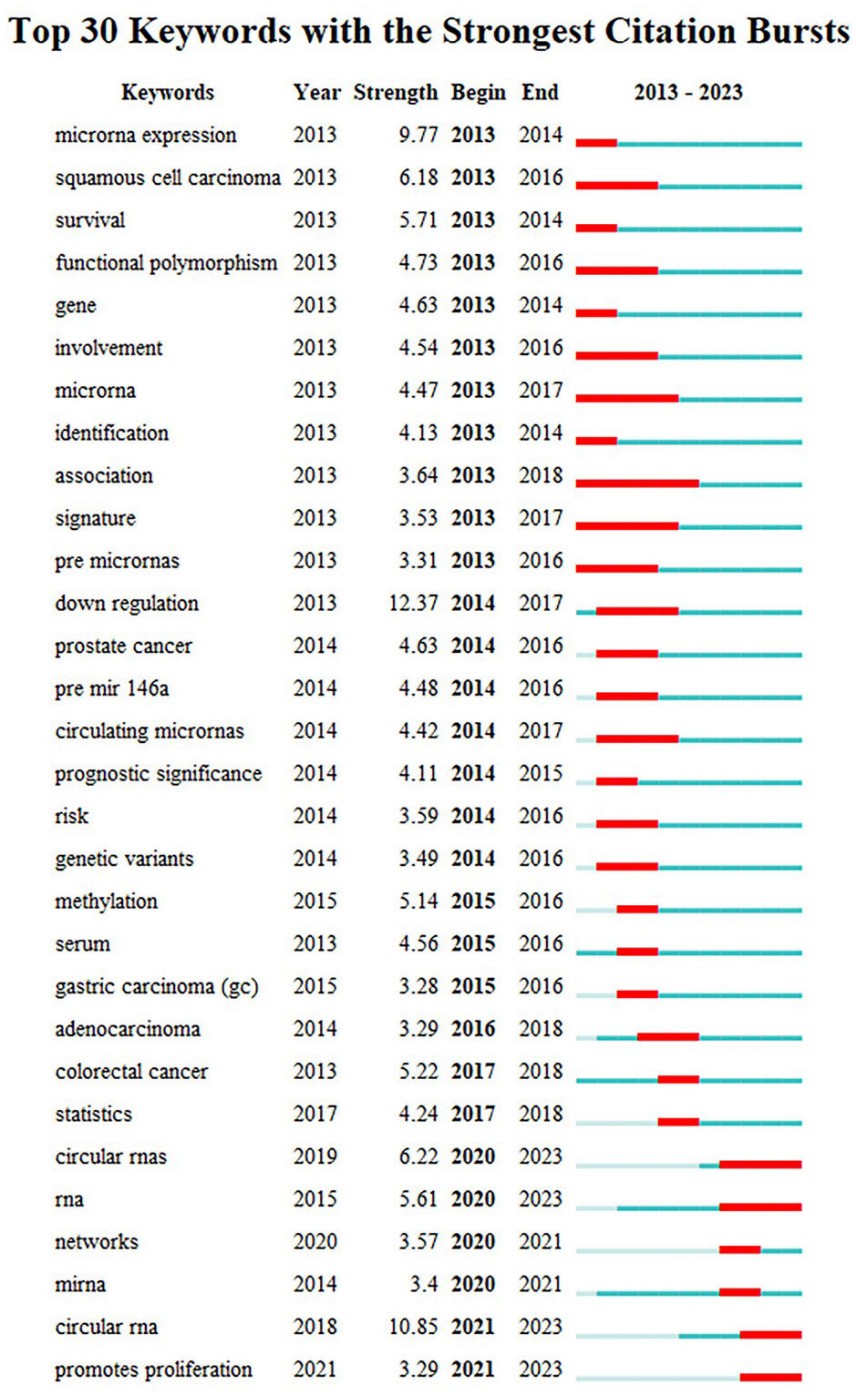
Top 30 keywords with the strongest citation bursts from 2013 to 2023. Note: “Strength” represents the citation bursts intensity of the keyword, a higher value indicates a higher frequency of occurrence during this period. The light blue line represents time period with lower frequency, and blue line indicates more frequent time period, marking it in red represents that the keyword is in the most frequent time period.

Burst keywords displayed large changes of the keywords in a short period, which helps to identify the research frontiers of a field. Among the top 30 keywords with the strongest citation bursts (**Figure 4**), “down regulation” was the keyword with the strongest burst (strength=12.37), followed by “circular RNA” (strength=10.85) and “microRNA expression” (strength=9.77). Besides, the keywords of “circular RNA”, “RNA”, and “promotes proliferation” have been bursting until 2023, and may be the hot topics for future research.

### 3.6 Co-citation timeline analysis

**Figure 5:**
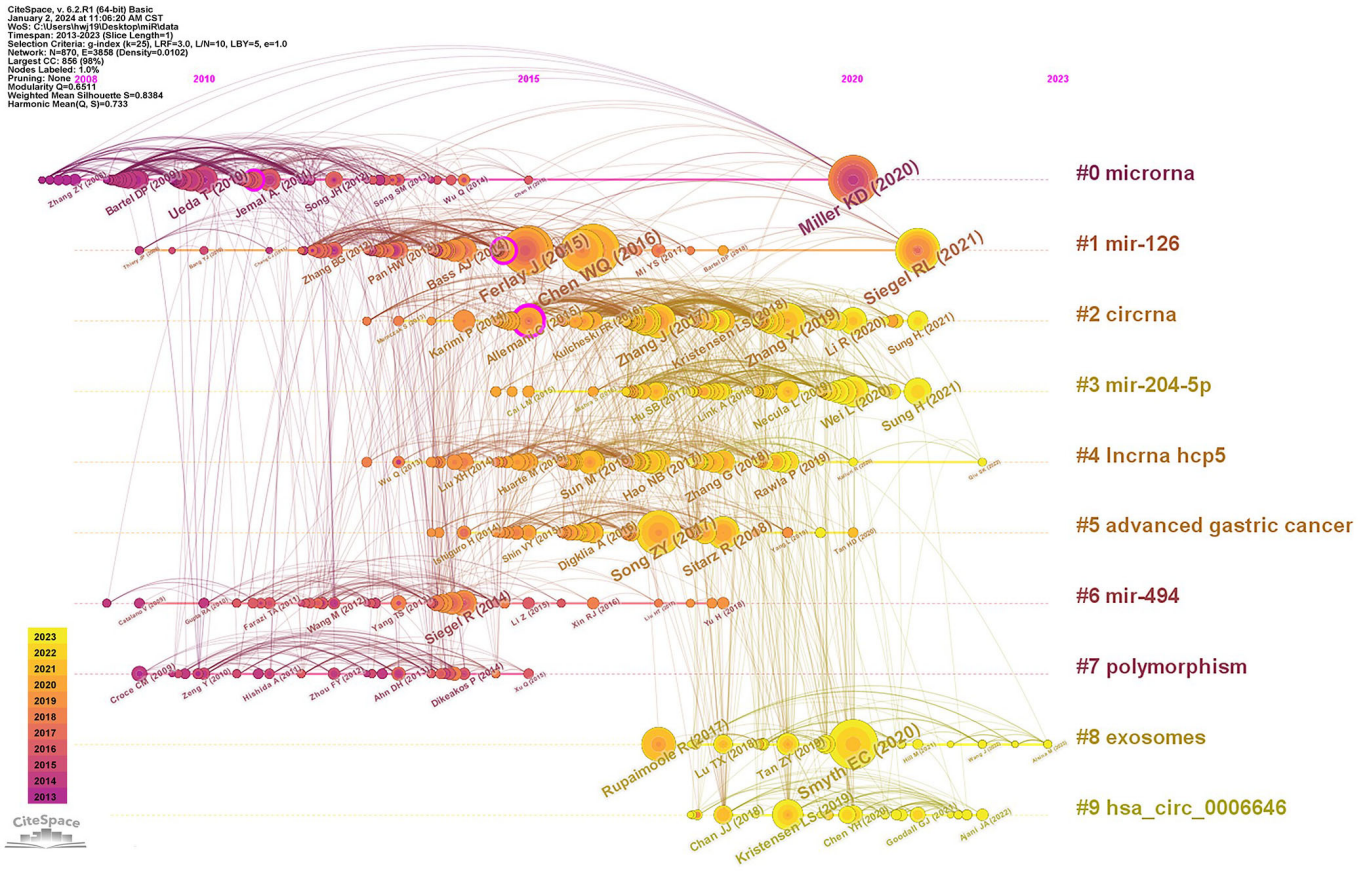
Timeline of co-citation clusters on miRNA in GC from 2013 to 2023.

Analysis of co-cited references can determine the core papers that plays a crucial role in a field, and keywords timeline analysis of reference co-citation can further reveal its changing trends of research hotspots (**Figure 5**). The network was divided into 10 clusters, with Q of 0.6511 and S of 0.8384. The largest cluster mainly included #0 microRNA, #1 mir-126, #2 circRNA, #3 mir-204-5p, #4 incRNA hcp5, #5 advanced gastric cancer, #6 mir-494, #7 polymorphism, #8 exosomes, #9 has_circ_0006646. It was evident that #6 mir-494 and #7 polymorphism were relatively early hotspots, and #3 mir-204-5p and #9 has_circ_0006646 were the most focused directions from 2015 to 2023. While #0 microRNA had always been research hotspot in the field of miRNAs in GC.

This paper also listed the 10 representative references in the field of miRNAs in GC based on citation frequency (**Table 3**). Ninety percent of the top 10 references were published in Q1, with 40% published in *CA-A Cancer Journal for Clinicians* (IF=254.7). Among the top 10 references, 7 were statistical analyses of cancer epidemiology (34–40), among which 2 papers (No.1 and No.2) (34, 37) published by Chen WQ, et al. and Miller KD, et al. have high contributions and recognition (citation count=134 and 116, centrality=0.04 and 0.06, respectively). The other 3 references were reviews on the treatment of GC (41–43), with two highlighting the current status of Circular RNA therapy in clinical practice (42, 43).

**Table 3:** The top 10 cited references on GC.

No.	Count	Centrality	Title	Author	Journal (IF^*^/JCR category)	Published year
1	134	0.04	Cancer Statistics in China, 2015	Chen Wangqing, et al.	Ca-A Cancer Journal for Clinicians (254.7/Q1)	2016
2	116	0.06	Cancer statistics, 2020	Rebecca L Siegel, et al.	Ca-A Cancer Journal for Clinicians (254.7/Q1)	2020
3	107	0.04	Cancer incidence and mortality worldwide: Sources, methods and major patterns in GLOBOCAN 2012	Jacques Ferlay, et al.	International Journal of Cancer (6.4/Q1)	2015
4	101	0.01	Gastric cancer	Elizabeth C Smyth, et al.	Lancet (168.9/Q1)	2020
5	95	0.03	Global Cancer Statistics, 2012	Lindsey A Torre, et al.	Ca-A Cancer Journal for Clinicians (254.7/Q1)	2015
6	92	0.09	Cancer Statistics, 2021	Rebecca L Siegel, et al.	Ca-A Cancer Journal for Clinicians (254.7/Q1)	2021
7	86	0.04	Gastric cancer	Eric Van Cutsem, et al.	Lancet (168.9/Q1)	2016
8	81	0.02	Progress in the treatment of advanced gastric cancer	Song Zheyu, et al.	Tumor Biology (N/A)	2017
9	59	0.01	Circular RNA circNRIP1 acts as a microRNA-149-5p sponge to promote gastric cancer progression via the AKT1/mTOR pathway	Zhang Xing, et al.	Molecular Cancer (37.3/Q1)	2019
10	55	0.04	Circular RNA_LARP4 inhibits cell proliferation and invasion of gastric cancer by sponging miR-424-5p and regulating LATS1 expression	Zhang Jing, et al.	Molecular Cancer (37.3/Q1)	2017

*The IF were obtained from the 2022 Journal Citation Reports (JCR).

## 4 Discussion

### 4.1 General information

By conducting a bibliometric and visual analysis of literature regarding miRNAs in GC over the last decade, tables and maps were generated to help reveal the research status, hotspots, and emerging trends. Overall, the annual publications in this field have gradually increased, reaching a peak in 2020. China is in a leading position in this field, as its number of publications is nearly 29 times that of the second ranked country, and the top 10 prolific institutions and authors are all from China. The reasons may include the following two aspects. First, 43.9% and 48.6% of the world’s new GC cases and cancer-related deaths occur in China (2), the incidence rate is increasing, and the disease burden is becoming heavier and heavier (44). It is urgent to find new targets and new methods to treat GC. Secondly, early detection and treatment are crucial for improving the survival rate of GC, and South Korea’s early diagnosis plan for GC has made it ranked first in the world in terms of five-year survival rate (45). While the screening rate for GC is relatively low in China, and the early diagnosis rate of GC is less than 20% (46). In addition to popularizing the health benefits of early screening for GC among residents to improve the early diagnosis rate, it is also particularly important to explore the evidence of multi-gene risk scoring to predict the genetic risk of GC in Chinese population (47), and to develop minimally invasive and reliable early screening tools. Interestingly, some countries with low outputs in this field, such as Germany and Canada, also have close cooperation relationships with other countries (centrality>0.1). After reviewing these publications, it was found that the authors of the same publications from different countries mostly came from the same research institutions or only participated in the revisions of the publications, without achieving true inter-country cooperation. Studies have shown that communication and cooperation between authors and institutions may be positively correlated with research productivity and research quality (48, 49), so it was recommended that researchers focused on this field strengthen their experience exchange and cooperation with authors who have published more papers, so as to promote the transformation of basic achievements into clinical drugs and the development of this field.

### 4.2 Research hotspots

Based on keyword co-occurrence clustering, as well as the frequency and centrality of keywords, this study summarized three research hotspots in this field. Firstly, the role of miRNAs as biomarkers in GC is a hot topic (50, 51). Many case-control studies have found that miRNAs [e.g., miR-21 (17, 52), miR-106 (53), miR-129, miR-196 (54), miR-214 (55), miR-135 (56), miR-223 (57), miR-421 (21), and miR-584 (58)] with different expression levels in GC patients have potential diagnostic and prognostic value for GC compared to healthy subjects. However, most of them had small sample sizes and different schemes for collecting and processing blood samples, resulting in poor repeatability of results (51, 59). Encouragingly, GASTRO Clear™, the world’s first serum GC molecular diagnostic panel based on 12-miRNAs, has been approved and launched in Singapore in 2019, with detection sensitivity and specificity reaching 87% and 93.9%, respectively (60), greatly enhancing the detection rate of stage I and II of GC. However, early trials of this panel only included Asians, and further validation is needed to determine whether it is applicable in other regions (60). It is recommended to conduct large-scale, multicenter prospective studies to develop multivariate detection schemes and validate existing results, so as to achieve the goal of early diagnosis of GC.

The second research hotspot is the mechanisms of miRNAs in the progression of GC. The STAT3 signaling pathway (61–63) and suppressor of cytokine signaling 3 (SOCS3) (64) have been found to be crucial for certain miRNAs that regulate the growth, apoptosis, proliferation, invasion, drug resistance, and immune-modulatory function of GC cells (e.g., miR-125a reduces the expression of STAT3 to inhibit the migration and metastasis of GC cells (61), miR-665 directly inhibits SOCS3 activation to promote the cell growth, proliferation, and migration of GC cells (64)). Besides, studies have shown that epithelial-to-mesenchymal transition (EMT) is associated with the invasiveness, metastasis, and drug resistance of GC cells (65, 66). P53 regulates EMT related transcription factors (e.g., snail and ZEB1/2) by transcriptionally controlling the expression levels of certain miRNAs (e.g., miR-130b) to promote EMT and cancer cell invasions (67, 68). In addition, SRY-Box Transcription Factor 4 (SOX4) is considered an important developmental transcription factor and an independent prognostic factor in human cancer, playing a crucial role in regulating EMT (69). Although there have been mounting studies exploring the regulatory pathways of miRNAs in GC progression and achieved remarkable results, transforming basic research results into clinical applications remains a challenge for future cancer research and drug development.

The third popular topic is the mutual effects between miRNAs and *Helicobacter pylori* (*H. pylori*) on GC. On the one hand, *H. pylori* can affect the expression levels of miRNAs by promoting DNA methylation (70, 71) and inducing or inhibiting the activation of transcription factors (58, 72), thereby promoting cell proliferation, inhibiting cell apoptosis, participating in the regulation of inflammatory processes, and regulating cell autophagy, invasion, and migration (73–75). On the other hand, miRNAs can in turn affect the colonization of *H. pylori* by altering host related metabolism and regulating the progression of GC induced by *H. pylori* (14, 76). However, in the past five years, researchers have focused more on the effects of *H. pylori* on the expression levels of miRNAs related to GC, there is still a lack of systematic understanding of the regulatory pathways and specific mechanisms of miRNAs on *H. pylori* (73, 77). In the future, it is recommended to strengthen interdisciplinary cooperation, such as the integration of tumor and immunology, to identify potential treatment targets and develop new treatment strategies.

### 4.3 Hotspots evolution and research frontiers

The analysis of burst keywords and the keywords timeline map of reference co-citation can reflect the development process and frontiers in a field. Before 2015, the researches devoted mainly to exploring the relationships between the polymorphisms of miRNAs and the risks of GC (78, 79), with mir-192 being the most popular sequence among potential tumor biomarkers. From 2015 to 2020, scholars paid more attention to Wnt/β-catenin signaling pathway and their effects on GC cells (80, 81), with mir-221-3p being the hot sequence regulating the progression of GC cells. In recent years, keywords such as “glucose metabolism”, “circular RNA”, and “sponge” have received high attention, highlighting the future directions and research frontiers of this field.

Glucose metabolism is an important pathway for GC cells to quickly obtain energy for proliferation and migration, which is closely related to the occurrence, development, and poor prognosis of GC (82–84). Blocking the glycolytic pathway can effectively inhibit the proliferation of GC cells and even kill them (85). However, the explorations of miRNAs and their pathways related to glucose metabolism in GC cells still face enormous challenges. Moreover, the regulatory roles of circular RNAs (circRNAs) as miRNAs sponges (86) on GC progression are gaining increasing research interests (87, 88), but there are limited preclinical studies on their application in GC treatment. In the future, further elucidations of the regulatory networks of the mutual effects of circRNAs and miRNAs on GC cells, as well as in-depth explorations of their mechanisms in GC progression are needed.

### 4.4 Strengths and limitations

As far as we know, this is the first bibliometric analysis to decipher the research status and trends of research on miRNAs in GC. This study, covering 11 years of research in this field, can empower scholars to obtain an intuitive overview and formulate novel research ideas, thereby advancing academic development of this field. However, the limitations of this study should also be considered. Firstly, only English literature during the period of 2013 to 2023 from the WoSCC database were extracted, which may result in non-comprehensive data for analysis. Secondly, this paper only retrieved the publications on “miR” and GC, which may not fully represent all studies on miRNAs in GC. However, the WoSCC database included the world’s most influential and prestigious academic journals, and the 2,051 publications analyzed in this study may reduce this bias and effectively represent the global status of this field.

## 5 Conclusion

This study provided a quantitative bibliometric analysis of literature on miRNAs in GC. The research hotspots included the roles of miRNAs as biomarkers in GC, the mechanisms of miRNAs in the progression of GC, as well as the mutual effects of miRNAs and *H. pylori* on GC. The mechanisms of miRNAs on glucose metabolism in GC and the application of sponge effect of circRNAs on miRNAs in GC treatment will likely be frontiers of future research. Furthermore, considering the regional differences in tumor immune markers, it is necessary to strengthen scientific research exchange and cooperation between institutions and authors in the same region, identify targets and therapeutic regimes for locally specific and high-risk GC types, and promote the transformation of research results into clinical practices.

## Author contributions

XW: Writing – original draft. CW: Writing – original draft. WH: Writing – review & editing. CM: Writing – review & editing. JS: Writing – review & editing. TW: Writing – review & editing. ZH: Writing – review & editing. SL: Writing – review & editing. RW: Writing – review & editing.
