# Supplementary material for: Bibliometric and visualized analysis of global research on microRNAs in gastric cancer: from 2013 to 2023
Source: Front Oncol. 2024 May 10;14:1374743. doi: 10.3389/fonc.2024.1374743 (PMC11116657; doi:10.3389/fonc.2024.1374743)
Supplement: Supplementary file 1 [file Table_1.docx]

Table S1. Top 10 most prolific journals on miRNA in GC.

| Rank | Journal | Number (%) | Country | IF^*^ | JCR quartile |
| --- | --- | --- | --- | --- | --- |
| 1 | Oncotargets and Therapy | 65(3.17) | England | 4.0 | Q2 |
| 2 | Oncotarget | 57(2.78) | United States | N/A | N/A |
| 3 | European Review for Medical and Pharmacological Sciences | 52(2.54) | Italy | 3.3 | Q2 |
| 4 | Cancer Cell International | 45(2.19) | England | 5.8 | Q1 |
| 5 | International Journal of Clinical and Experimental Pathology | 42(2.05) | United States | 1.4 | N/A |
| 6 | Oncology Letters | 40(1.95) | Greece | 2.9 | Q3 |
| 7 | Cell Death & Disease | 39(1.90) | England | 9.0 | Q1 |
| 8 | Tumor Biology | 38(1.85) | Switzerland | N/A | N/A |
| 9 | Cancer Management and Research | 35(1.71) | New Zealand | 3.3 | Q3 |
| 10 | Biomedicine & Pharmacotherapy | 33(1.61) | France | 7.5 | Q1 |

Notes: *The IF were obtained from the 2022 Journal Citation Reports (JCR).
